# Fast Evolution from Precast Bricks: Genomics of Young Freshwater Populations of Threespine Stickleback *Gasterosteus aculeatus*

**DOI:** 10.1371/journal.pgen.1004696

**Published:** 2014-10-09

**Authors:** Nadezhda V. Terekhanova, Maria D. Logacheva, Aleksey A. Penin, Tatiana V. Neretina, Anna E. Barmintseva, Georgii A. Bazykin, Alexey S. Kondrashov, Nikolai S. Mugue

**Affiliations:** 1 Department of Bioinformatics and Bioengineering, M. V. Lomonosov Moscow State University, Moscow, Russia; 2 A. N. Belozersky Institute of Physico-Chemical Biology, M. V. Lomonosov Moscow State University, Moscow, Russia; 3 Department of Genetics, Biological faculty, M. V. Lomonosov Moscow State University, Moscow, Russia; 4 White Sea Biological Station, Biological faculty, M. V. Lomonosov Moscow State University, Moscow, Russia; 5 Laboratory of Molecular genetics, Russian Institute of Fisheries and Oceanology, Russian Federal Research Institute of Fisheries and Oceanography, Moscow, Russia; 6 Sector for Molecular Evolution, Institute for Information Transmission Problems of the RAS (Kharkevich Institute), Moscow, Russia; 7 Department of Ecology and Evolutionary Biology and Life Sciences Institute, University of Michigan, Ann Arbor, Michigan, United States of America; 8 N. K. Koltsov Institute of Developmental Biology RAS, Moscow, Russia; Fred Hutchinson Cancer Research Center, United States of America

## Abstract

Adaptation is driven by natural selection; however, many adaptations are caused by weak selection acting over large timescales, complicating its study. Therefore, it is rarely possible to study selection comprehensively in natural environments. The threespine stickleback (*Gasterosteus aculeatus*) is a well-studied model organism with a short generation time, small genome size, and many genetic and genomic tools available. Within this originally marine species, populations have recurrently adapted to freshwater all over its range. This evolution involved extensive parallelism: pre-existing alleles that adapt sticklebacks to freshwater habitats, but are also present at low frequencies in marine populations, have been recruited repeatedly. While a number of genomic regions responsible for this adaptation have been identified, the details of selection remain poorly understood. Using whole-genome resequencing, we compare pooled genomic samples from marine and freshwater populations of the White Sea basin, and identify 19 short genomic regions that are highly divergent between them, including three known inversions. 17 of these regions overlap protein-coding genes, including a number of genes with predicted functions that are relevant for adaptation to the freshwater environment. We then analyze four additional independently derived young freshwater populations of known ages, two natural and two artificially established, and use the observed shifts of allelic frequencies to estimate the strength of positive selection. Adaptation turns out to be quite rapid, indicating strong selection acting simultaneously at multiple regions of the genome, with selection coefficients of up to 0.27. High divergence between marine and freshwater genotypes, lack of reduction in polymorphism in regions responsible for adaptation, and high frequencies of freshwater alleles observed even in young freshwater populations are all consistent with rapid assembly of *G. aculeatus* freshwater genotypes from pre-existing genomic regions of adaptive variation, with strong selection that favors this assembly acting simultaneously at multiple loci.

## Author Summary

Adaptation to novel environments is a keystone of evolution. There is only a handful of natural and experimental systems in which the process of adaptation has been studied in detail, and each studied system brings its own surprises with regard to the number of loci involved, dynamics of adaptation, extent of interactions between loci and of parallelism between different adapting populations. The threespine stickleback is an excellent model organism for evolutionary studies. Marine-derived freshwater populations of this species have consistently acquired a specific set of morphological, physiological and behavioral traits allowing them to reside in freshwater for their whole lifespan. Previous studies identified several genomic regions responsible for this adaptation. Here, using whole-genome sequencing, we compare the allele frequencies at such regions in four derived freshwater populations of known ages: two natural, and two artificially established in 1978. Knowledge of population ages allows us to infer the strength of selection that acted at these loci. Adaptation of threespine stickleback to freshwater is typically fast, and is driven by strong selection favoring pre-existing alleles that are likely present in the ancestral marine population at low frequencies; however, some of the adaptation may also be due to young population-specific alleles.

## Introduction

Studies of adaptation in nature are complicated by the typically long timescales at which evolution proceeds, and therefore are rather rare (e.g. [1]–[3]). Positive selection, the hallmark of adaptation, can be inferred from patterns of divergence and/or polymorphism in genome comparisons. While experimental evolution coupled with searches for patterns consistent with positive selection is becoming an accepted tool for “real time” studies of adaptation in microbes [4], [5], it is rarely possible to use genomic data to observe the adaptation process in higher animals such as vertebrates [6], [7]. Furthermore, the mechanisms of adaptation at the genomic level are still poorly understood [8]–[10].

The study of the genomics of adaptation has experienced a recent upheaval since the advent of population-level next-generation sequencing, which enables identification of selected loci and detailed studies of divergence and polymorphism within them in a wide range of model systems [11]–[13]. The data reveal that the number of loci responsible for adaptation, the ratio of coding and regulatory changes, the proportions of parallel to non-parallel genetic changes vary between systems [13]–[15]. The reasons for such variation are still unclear, making further genomic studies of adaptation a priority.

Threespine stickleback (*Gasterosteus aculeatus*) has become a widely used model organism for studying adaptation and speciation [16], [17]. The species is very variable, and is represented by a number of morphs [18], [19]. The ancestral populations of *G. aculeatus* likely lived in the sea, and colonization of new freshwater habitats, followed by evolution of freshwater populations, occurred repeatedly all over the Northern Hemisphere. While fish from marine populations utilize freshwater lakes and streams only as temporary spawning grounds, thousands of isolated freshwater resident populations have been independently established, and they have diverged in morphological, physiological and behavioral traits allowing them to survive in the freshwater for their entire lifespan. Independent origin of freshwater populations of *G. aculeatus* in different locations in the Northern Hemisphere provides an opportunity to study adaptive evolution under similar environments [15], [20]–[24]. Much of this adaptive evolution has been shown to be parallel, involving repeated recruitment for adaptation at different freshwater populations of the same pre-existing alleles that are presumably carried at low frequencies by marine populations. However, some of the adaptations are specific to individual populations [15],[22]; the relative importance of adaptations by new mutations vs. standing variation, and of population-specific vs. parallel adaptations, is not known.

Freshwater and marine forms of *G. aculeatus* possess a number of phenotypic differences. One of the most obvious is their armor plates: while the marine form has a complete set of lateral plates covering their body from pelvic girdle to the caudal peduncle, there are usually just a few lateral plates in the freshwater form [25],[26]. Genetic differences responsible for the number of the armor plates have been identified, pointing to the *EDA* gene on chromosome IV [27]. Later, sequencing of the *G. aculeatus* genome (available at http://genome.ucsc.edu) facilitated studying the genetic basis of the differences between the two forms, and several large genomic regions with high concentrations of nucleotide substitutions between the forms were found by comparing individuals from marine and freshwater habitats in a RAD-Seq analysis [22]. A recent study of *G. aculeatus* from Atlantic and Pacific basins used whole genome sequencing to reveal more than two hundred small genetic regions throughout the stickleback genome that differ between the forms [15].

Populations of *G. aculeatus* adapted to freshwater inhabit lakes and streams that originated after the retreat of the Pleistocene glaciers, indicating that adaptation can be fast [16]. Although rather different phenotypically, the freshwater and the marine forms often can hybridize and produce fertile offspring [18],[28],[29]. However, in some populations, there can also be a nearly-complete reproductive isolation in natural habitats between freshwater residential populations and anadromous marine forms spawning in the same lake [30],[31]. Reproductive isolation is mediated by phenotypic traits [32], and generally, there is not a clear cut relationship between the age of freshwater populations and reproductive isolation between marine and freshwater morphs.

Studies of *G. aculeatus* in the White Sea and its basin were initiated by Valery Ziuganov in the 1970s [33],[34]. The upper boundary for the age of the marine population in the White Sea is 15,000–18,000 years, because earlier, this area was covered in ice sheets during the Last Glacial Maximum [35]. After the end of the glaciation, the White Sea region experienced eustatic raising, giving rise to a unique system of young lakes as bays gradually separated from the sea by lift of the coast [36]. The rate of this process, which is still ongoing, has been estimated as 3.8 mm/year [37]; this allows inferring the age of a freshwater population from the elevation of the lake above sea level. Furthermore, in 1978, Ziuganov established several independent artificial sticklebacks populations in abandoned mica and spar quarries filled with ground water, by seeding each quarry with controlled numbers of marine and freshwater individuals [33]. Sampling these populations in 2011 allowed us to study two evolutionary trajectories with known points of departure. Thus, the availability of a wide range of young lakes of known ages in the White Sea basin provides an opportunity to trace the dynamics of adaptation to freshwater environments.

Here, we use whole-genome sequencing to study eight populations of *G. aculeatus* from the White Sea basin, including two artificial populations. We aimed to detect the genetic differences between the ancestral marine and the derived freshwater populations, and to measure the rate of adaptation, and the strength of positive selection which drives it, at divergent genomic loci. Whole-genome comparisons of multiple artificial and natural derived populations allow detailed analysis of selection acting simultaneously at multiple loci. Using multiple populations of different ages allows studying the process of adaptation at these loci at a range of time points, from tens to hundreds of years, and the uniformity of the process of selection. Finally, whole-genome analysis reveals the detailed patterns of divergence and polymorphism within the selected loci and in their vicinity.

## Results

### Genetic differences between the marine and the freshwater populations

**Figure 1:**
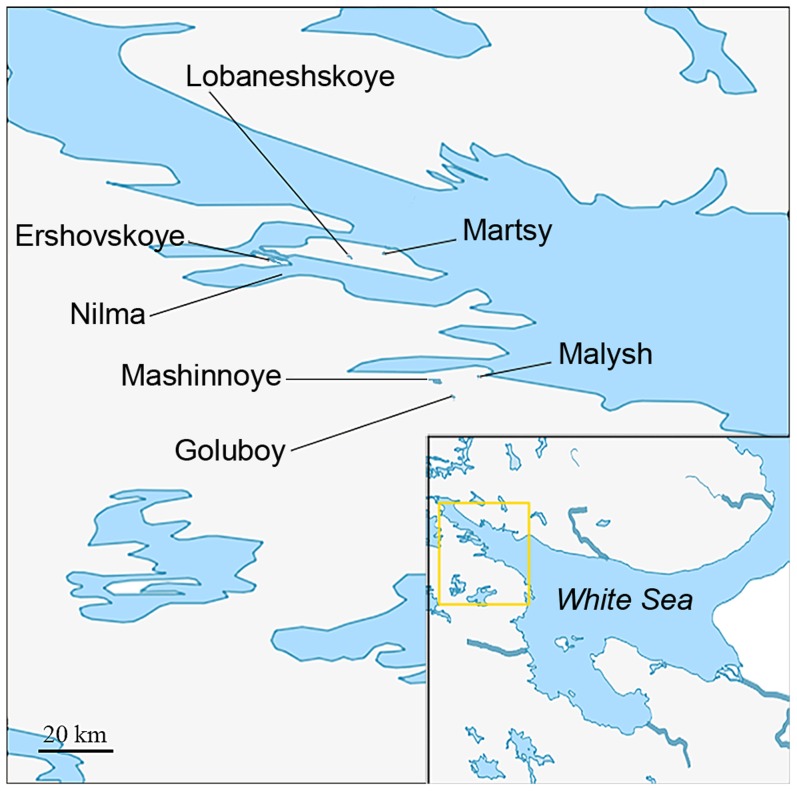
Map showing the locations of populations studied. Please see Table 1 for description of sampling sites.

We searched for the genetic markers of differences between the ancestral population of *G. aculeatus* in the White Sea and the derived freshwater populations in its vicinity. To identify such markers, we compared the genome sequences of two samples of marine individuals with two samples of freshwater individuals (Figure 1, Table 1). Phenotypically marine individuals were collected in Nilma bay and among the anadromous (marine) fish in Lake Ershovskoye where they came to spawn. Phenotypically freshwater individuals were collected from Lake Lobaneshskoye on the Island Velikiy and Lake Mashinnoye on the mainland. Their ages since desalination, inferred from their current elevations above the sea level, are ∼600 and ∼700 years, respectively [37]. By using these two freshwater populations of independent origins which are the oldest in the area, we aimed to identify those genetic changes that occurred in parallel in both freshwater populations, and therefore likely include sites responsible for adaptation of *G. aculeatus* to freshwater.

**Table 1:** Sampling sites and library characteristics.

Sample	Description	Geographic location	No. individuals sampled	Library length, bp	Number of reads	Number of reads mapped	Mean coverage, fold	Nucleotide diversity (π)
Nilma^a^	Anadromous fish sampled from the sea	66°30.45′N, 33°7.68′E	16	571	228,077,463	204,905,523	44	0.00210
Ershovskoye (anadromous)^a^	Anadromous fish sampled from the Ershovskoye lake	66°32.21′N, 33°3.62′E	10	462	90,133,314	80,480,814	17	0.00188
Ershovskoye (residential)^b^	Freshwater lake, estimated age 33 years [33]	66°32.21′N, 33°3.62′E	12	538	275,789,662	241,741,391	52	0.00219
Martsy^b^	Freshwater lake, estimated age ∼250 years	66°35.95′N, 33°15.35′E	10	550	136,851,580	111,360,705	24	0.00181
Goluboy^c^	Quarry, population seeded in 1978 with 20 anadromous and 20 freshwater fish [34]	66°17.20′N, 33°23.29′E	20	572	261,538,220	224,807,642	48	0.00204
Malysh^c^	Quarry, population seeded in 1978 with 1 anadromous and 1 freshwater fish [34]	66°18.27′N, 33°25.27′E	20	613	334,921,323	293,874,942	63	0.00186
Lobaneshskoye^d^	Freshwater lake, estimated age ∼600 years	66°33.64′N, 33°13.45′E	8	572	126,403,232	107,371,357	23	0.00167
Mashinnoye^d^	Freshwater lake, estimated age ∼700 years	66°17.74′N, 33°21.82′E	10	512	110,084,409	87,460,315	19	0.00158

a marine population;

b recent natural freshwater populations;

c artificial freshwater populations;

d older natural freshwater populations.

Columns provide description, coordinates of the sampling sites, number of individuals in the sample, characteristic of the libraries, numbers of reads, coverage and nucleotide diversity for each sample.

We estimated allele frequencies from pooled samples of individuals; these allele frequencies were confirmed using allele-specific PCR for specific loci (see below). We defined “marker” single-nucleotide polymorphisms (SNPs) as polymorphic nucleotide sites where both marine samples contained a particular allele at frequencies above 80%, while both freshwater samples contained another allele at frequencies above 80% (“strong criterion”) or above 50% (“weak criterion”). For comparison, we also identified marker SNPs according to the strong criterion using only one marine-freshwater pair of populations (Nilma vs. Mashinnoye).

**Figure 2:**
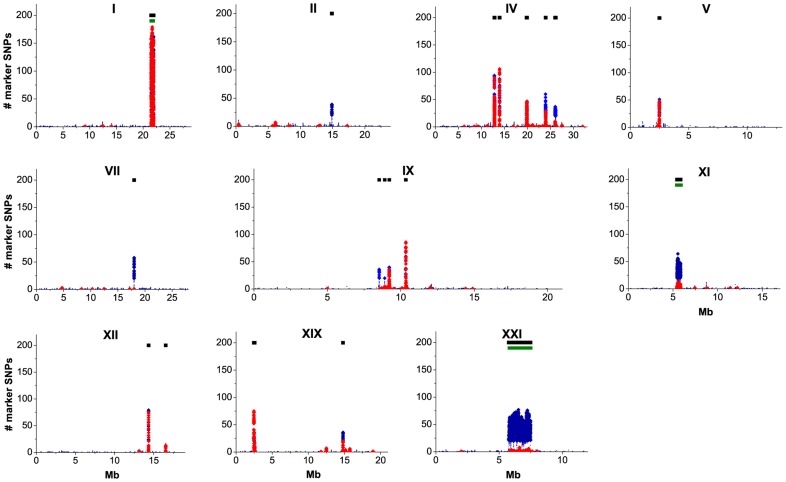
Distribution of SNPs that distinguish the marine and freshwater populations along the *G. aculeatus* genome. For each chromosome carrying a DI of marker SNPs, the horizontal axis shows the position along the chromosome in Mb and the vertical axis shows, for 10 Kb frames, the numbers of marker SNPs under the strong (red) or weak (blue) criterion. To reduce clutter, only every 1000th frame is displayed, and the numbers of weak marker SNPs below 20 are represented by small dots. Black bars identify DIs of marker SNPs, and green bars at chromosomes I, XI and XXI underlie known inverted genome segments.

Identified marker SNPs were distributed unevenly along the reference genome, clearly consisting of dense aggregations (“divergence islands”, DIs; [11],[15]) of markers in short genomic regions. A strong (weak) DI was defined as a continuous region where each 10 Kb window carried at least 10 strong (20 weak) markers, after merging any two such regions that are closer than 40 Kb to each other, because recombination is not likely to occur on such short distance [38],[39]. This definition, which seems to describe our data well (Figure 2), leads to a smaller number of wider DIs than the definitions used in [15] (see Methods for an alternative approach). Among the 6,107 marker SNPs obtained under the strong criterion, 5,801 (95.0%) were concentrated in DIs. By overlapping the strong and the weak criteria, we identified 19 DIs, which were located on ten out of the 21 *G. aculeatus* chromosomes (Figure 2) and covered a total of 3,301,948 nucleotides, or 0.74% of the genome.

In the majority of the DIs, the number of weak markers is only slightly above the number of strong markers, indicating that the freshwater-specific alleles have usually reached the frequency of 80% in both lake populations. Two exceptions are DIs IV-5 and XI-1 (i.e., the fifth DI on the 4^th^ chromosome, and the first DI on the 11^th^ chromosome), which, although identifiable both by the strong and the weak criteria, contains twenty times as many weak markers as strong markers. Furthermore, five DIs (II-1, VII-1, IX-1, IX-2 and XXI-1) could be identified only by the weak criterion, indicating that the frequency of freshwater-specific alleles in one or both freshwater populations is below 0.8.

The overall nucleotide diversity within the two freshwater samples was reduced by 22% relative to that in the two marine samples (Table 1), consistent with lower effective sizes of freshwater populations and/or moderate bottlenecks in the course of their origin. Both strong and weak DIs in marine population carried higher levels of nucleotide diversity (0.0049 and 0.0034, respectively), compared to the genomic background (0.0020). In the freshwater populations, diversity was reduced in the strong DIs (0.0012), but elevated in the weak DIs (0.0048), compared to the genomic background (0.0016).

Seventeen out of the 19 detected DIs overlap protein-coding genes, for a total of 170 genes (Table 2, Table S1); among the 285 marker SNPs identified under the strong marker criteria and covered by these genes, 139 were nonsynonymous, while 146 were synonymous (Table 3). The ratio of nonsynonymous to synonymous marker SNPs was much higher than that within non-marker SNPs in protein-coding genes segregating within the marine population (290 nonsynonymous to 528 synonymous; Table 3), implying positive selection favoring the preferential fixation of amino-acid changing marker SNPs between marine and freshwater populations [40]. However, 7 of the 17 DIs do not include any nonsynonymous marker SNPs. The remaining two DIs do not overlap any known protein-coding or miRNA genes. The DIs encompass genes that might affect several traits responsible for phenotypic difference between the marine and freshwater forms, including the well-known *EDA* gene responsible for body armor ([27],[41], DI IV-1), as well as genes likely to be important for adaptation to freshwater through their effects on osmoregulation, immunity, or morphology: Na+/K+ transporting ATPase (*ATP1A1* [42], DI I-1), neurotransmitter and hormone binding (*SULT4A1* [43], DI IV-4), and immunity response to viral infection (*NLRC5* [44], DI XIX-1). Other genes might be involved in several important aspects of metabolism and behavior (*INHA* [45], DI I-1), responsible for growth and development of nerve cells (*SLITRK2* [46], DI IV-5), adhesion and differentiation of nerve cells (*CTNNA2* [47], DI IX-3), calcium/phosphate homeostasis (*STC2* [48], DI IV-2), and mediation of functions in the central and peripheral nervous systems (*HTR3A* [49], DI V-1).

**Table 2:** 19 DIs of marker SNPs distinguishing marine and freshwater populations.

DI	Start	End	No. of marker SNPs	Length	Also found in		No. of genes overlapping DI	No of ns SNPs	s_E_	s_G_
					Ref. 22	Ref. 15				
I-1	21,487,998	21,960,119	4,186	472,122	**yes**	**yes**	27	100	0.247	0.212
II-1*	14,874,366	14,898,826	73	24,461	yes	yes	0	0	0.115	0.146
IV-1	12,803,780	12,881,296	285	77,517	**yes**	**yes**	7	19	0.188	0.094
IV-2	13,930,002	13,959,331	168	29,330		yes	2	1	0.218	0.107
IV-3	19,811,922	19,914,666	209	102,745	**yes**	**yes**	6	0	0.212	0.163
IV-4	23,954,634	23,981,981	48	27,348	**yes**	**yes**	3	1	0.227	0.1
IV-5*	26,016,955	26,166,536	252	149,582	yes	yes	4	1	0.196	0.036
V-1	2,482,209	2,501,295	65	19,087		yes	2	3	0.255	0.239
VII-1*	17,982,351	18,002,671	84	20,321	**yes**	**yes**	4	6	<0	0.273
IX-1*	8,521,935	8,537,559	44	15,625		yes	2	0	0.062	0.026
IX-2*	8,901,816	8,910,115	20	8,300		yes	2	0	0.108	0.023
IX-3	9,208,158	9,227,809	46	19,652		yes	1	0	0.198	0.131
IX-4	10,334,101	10,353,801	114	19,701		yes	1	0	0.141	0.101
XI-1*	5,445,757	5,855,124	1,237	409,368	**yes**	yes	21	10	0.054	0.147
XII-1	14,338,229	14,358,336	91	20,108	yes	yes	2	0	0.023	0.193
XII-2	16,522,028	16,538,810	24	16,783		yes	2	0	0.162	
XIX-1	2,449,903	2,581,858	277	131,956	yes	**yes**	5	11	0.178	0.131
XIX-2	14,787,904	14,799,088	21	11,185	yes	**yes**	0	0	0.117	0.056
XXI-1*	5,759,879	7,486,635	6,900	1,726,757	**yes**	yes	79	63	0.177	<0
Total			14,144	3,301,948	12	19	170	215		

The divergence island IDs are in the format ‘chromosome number-DI number’. Asterisks denote DIs identified under the weak criteria; the remaining DIs are identified under the strong criteria. Bold font denotes regions identified in Refs 15 and 22 by the stringent criteria; non-bold font denotes regions identified in Refs 15 and 22 by less stringent criteria, and reported in supplementary materials for these refs. ns, number of nonsynonymous substitutions, *s*_E_, selection coefficient in favor of freshwater allele estimated from Lake Ershovskoye; *s*_G_, selection coefficient in favor of freshwater allele estimated from Quarry Goluboy.

**Table 3:** SNPs in protein-coding genes.

	Marine-freshwater			Within marine		
	Nonsynonymous	Synonymous	Ratio	Nonsynonymous	Synonymous	Ratio
Within DIs	139	146	0.95	290	528	0.55
Outside DIs	17	4	4.25	28,616	41,902	0.68

The numbers of nonsynonymous and synonymous SNPs within DIs and outside them, in the comparison of two marine and two freshwater populations (marker SNPs, under the strong criterion) and SNPs within the Nilma marine population.

Only 5.0% of marker SNPs identified under the strong marker criteria were not located within any of the DIs. This amounted to a total of 306 marker SNPs, located on 19 out of the 21 chromosomes. 21 of the 306 SNPs were located within protein-coding regions; of these 21 SNPs, 17 were amino-acid changing, while only 4 were synonymous. Again, this ratio of nonsynonymous to synonymous substitutions is higher than that observed within a single (marine) population (28,616 and 41,902, respectively; Table 3), consistent with positive selection favoring amino acid-changing mutations even outside the DIs. Notably, among the 12 genes that carried amino-acid changes, a number could be plausibly held responsible for adaptation and speciation in *G. aculeatus*. For example, *GCNT3* gene, which plays an important role in biosynthesis of mucin which is used for nest building [50], carries 4 marker SNPs, all nonsynonymous. *MUC*-like gene on the chromosome II carries 2 non-synonymous substitutions. Two nonsynonymous non-DI marker SNPs are positioned within 300 bp of the *EPX*-like gene on chromosome XIII; *EPX* is a gene contributing to the activity of eosinophils which are responsible for lysis of parasites [51]. Another nonsynonymous SNP is in *INSR* gene on the chromosome III; this gene is known to play a crucial role in early development and growth, and in the development of the neural system [52],[53].

### Dynamics of adaptation of *G. aculeatus* to freshwater

In addition to lakes Mashinnoye and Lobaneshskoye, we also sampled *G. aculeatus* from four bodies of freshwater of more recent origin: quarries Goluboy and Malysh, and lakes Martsy and Ershovskoye (Table 1), all located near the White Sea. Populations in Goluboy and Malysh were established by Ziuganov in 1978 in isolated pools that developed in quarries after they had been abandoned, but were, before the start of the experiments, devoid of fish; therefore, they both were 34 years old at the time of sampling. The Quarry Goluboy population (area ∼70,000 m^2^, carrying capacity over 1,000 fish) was started from 20 marine (10 females and 10 males) and 20 freshwater (10 females and 10 males) individuals. The Quarry Malysh population (∼75 m^2^, carrying capacity about 100 fish, but the number of reproducing males may be limited by the very low number of nesting sites) was started from 1 marine female and 1 freshwater male [34]. Founding marine individuals were taken from the White Sea, and founding freshwater individuals were taken from Lake Mashinnoye. Lakes Martsy and Ershovskoye originated through isolation of marine bays due to the steady glacio-isostatic rise of the coast at the rate of ∼3.8 mm per year [37]. The age of the freshwater population in Lake Martsy can be estimated as ∼250 years, because the surface of the lake is currently at about 1 meter above the sea level. As recently as in 1978, Lake Ershovskoye (now ∼14 cm above spring-tide level) was a typical meromictic lake, inhabited by only anadromous fish with the typical marine phenotype [33]. This lake became fresh soon afterwards, and now contains an abundant residential population, which can be easily distinguished from anadromous individuals both morphologically and by a different parasite load (*Schistocephalus solidus* are dominant in residential individuals, and nematodes in anadromous individuals [33]); therefore, we estimate the age of Lake Ershovskoye also as 34 years. Thus, at these four bodies of water, the adaptation of *G. aculeatus* populations to freshwater is likely to still be ongoing.

**Figure 3:**
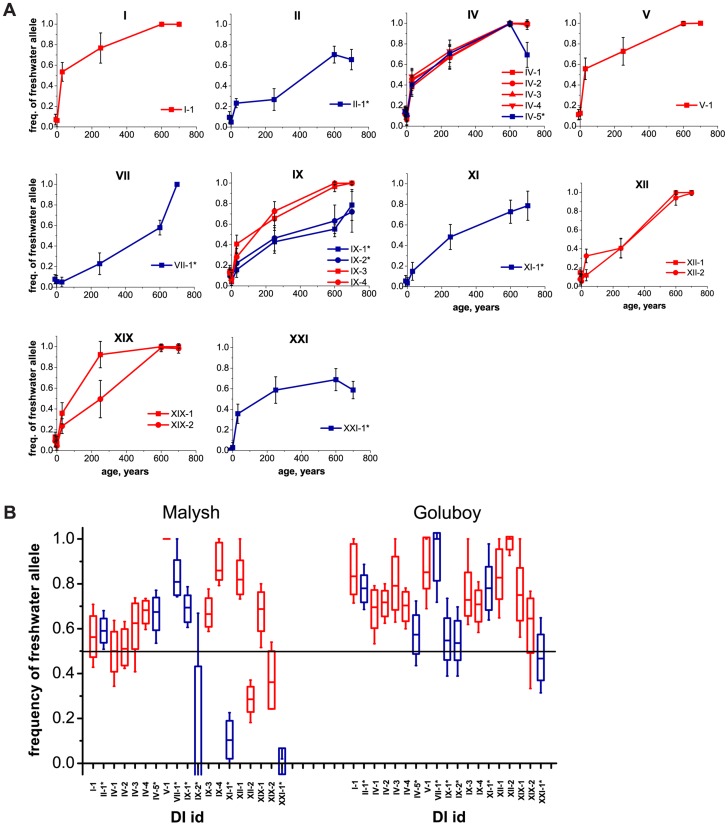
Mean frequencies of freshwater alleles at marker SNPs within identified DIs, at freshwater populations of different ages. (**A**) Natural populations. The horizontal axis shows the approximate ages of populations, ranging (left to right) from two marine populations (∼0 years) to recent (∼34 and ∼250 years) and older lake populations (∼600 and ∼700 years). Whiskers, standard deviation. (**B**) Experimental populations. For each of the DIs of marker SNPs, the assumed initial 50% frequency of freshwater alleles (black line) and their current frequencies are shown for two experimental populations: quarries Malysh (left) and Goluboy (right), each started in 1978. Dashes, boxes and whiskers correspond to the median, standard deviation, and 5^th^ and 95^th^ percentiles, respectively. Red, DIs identified under the strong criterion; blue, DIs identified under the weak criterion.

In all four young populations, frequencies of freshwater alleles at marker SNPs within DIs have been increasing rapidly (Figure 3, see Table S2 for allele frequencies data on all populations). For five of the DIs, the estimates of allele frequency obtained by Illumina sequencing of DNA pools were also validated by genotyping individual fish from each population with allele-specific primers (Table S3, Table S4). These increases imply that in each of the freshwater populations, selection favors the identified freshwater alleles. The initial frequencies of freshwater alleles in natural lakes Ershovskoye and Martsy were likely the same as the frequency observed in the marine population, i.e., ∼0.1 (Table S2). In the two quarry populations, the initial frequencies were assumed to equal 0.5. The mean frequency of freshwater alleles over all DIs in the artificial populations was 0.56 at Quarry Malysh, and 0.73 at Quarry Goluboy (Figure 3B). A lower average and a higher variance of freshwater allele frequencies at Quarry Malysh population are likely due to its small effective population size, and therefore, stronger genetic drift. The frequencies of freshwater alleles in Lake Martsy are higher than in Lake Ershovskoye, consistent with the former being older than the latter.

**Figure 4:**
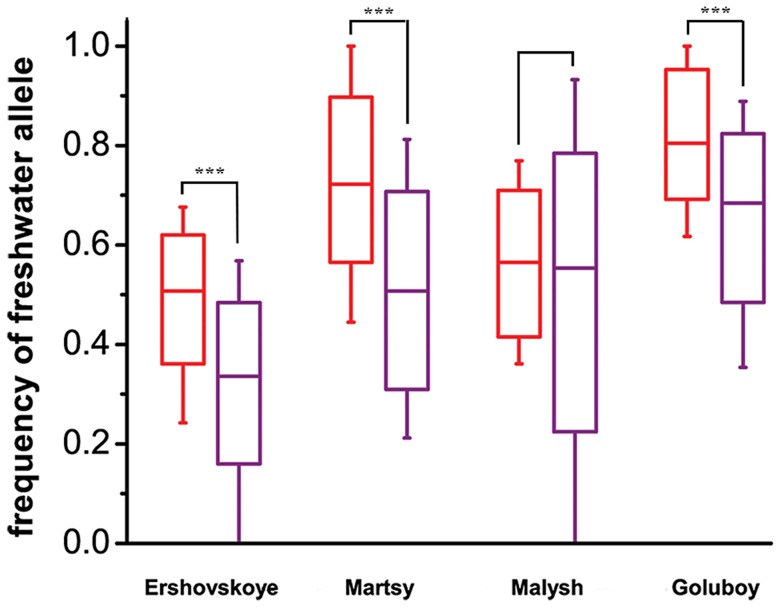
Comparison of mean frequencies of freshwater alleles at marker SNPs within and outside of identified DIs, at freshwater populations of different ages. Each two boxes correspond to the four young freshwater populations (anadromous from Lake Ershovskoye, Lake Martsy, Quarry Malysh, Quarry Goluboy). Statistical analysis was performed with two-tailed nonparametric Mann–Whitney *U* test. P values of <0.001 are designated with three (***) asterisks. Dashes, boxes and whiskers correspond to the median, standard deviation, and 5^th^ and 95^th^ percentiles, respectively. Red, marker SNPs (under the strong criterion) located within clumps; purple, marker SNPs (under the strong criterion) located outside clumps.

Freshwater alleles also increased in frequency at the marker SNPs located outside DIs. This was observed for all chromosomes in the two natural populations, except chromosomes XIV and XVI, each carrying only one marker SNP (Figure S1A); and for most of the chromosomes in the Quarry Goluboy artificial population (Figure S1B). Overall, however, the marker SNPs located within DIs have reached substantially higher frequencies in all freshwater populations, compared with the marker SNPs outside DIs (Figure 4). An increase of the frequency of freshwater alleles was also observed when only one marine-freshwater pair of populations (Nilma vs. Mashinnoye), rather than two pairs, was used to define the marker SNPs, although it was even less pronounced (Figure S3).

### Strength of selection responsible for adaptation

Knowing the rate of increase of an allele frequency makes it possible to estimate the strength of positive selection favoring this allele [54]. Because the age of the Lake Martsy is known only approximately, we cannot reliably estimate the rate of allele frequency change in it, while for the Lake Ershovskoye, the age of the population is known rather precisely. Furthermore, among the two artificial populations, selection in Quarry Goluboy was more pronounced than in Quarry Malysh, probably due to a stronger contribution of drift in the latter (see above). Therefore, for estimation of selection strengths, we used Lake Ershovskoye and Quarry Goluboy populations. We made such estimates assuming that the generation time of *G. aculeatus* is two years ([1] and our data; see Methods). We also made the simplifying assumptions that selection remained constant, and that freshwater alleles possess intermediate dominance (h = 0.5), as has been recently shown to be true, in particular, for most skeletal quantitative trait loci (QTL) in *G. aculeatus* [55].

We estimated the selection coefficient *s* for each DI (no estimates were made for non-DI marker SNPs, as estimates based on individual SNPs are unlikely to be robust). Only one selection coefficient *s* was ascribed to a DI, on the basis of the average frequency of freshwater marker alleles in it. As the allele frequencies within a DI are non-independent, the strength of selection cannot be estimated with precision, and the estimates for individual DIs should be treated with caution; still, this analysis allows us to appreciate the selection in effect.

**Figure 5:**
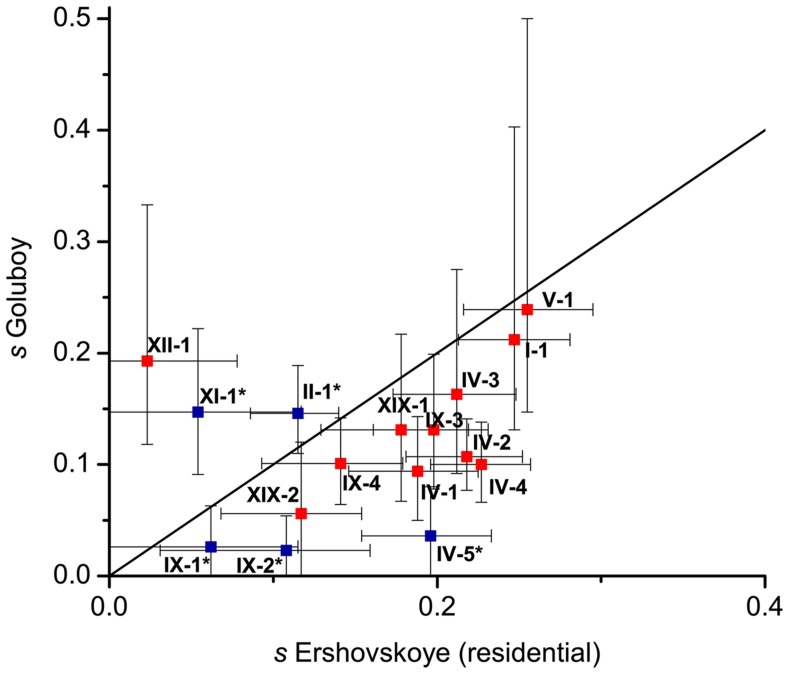
Selection coefficients at DIs estimated from Lake Ershovskoye and Quarry Goluboy. Red, DIs identified under the strong criterion; blue, DIs identified under the weak criterion. Three DIs are not shown: VII-1 and XXI-1, which had inferred *s* below 0 in one of the populations; and XII-2, for which *s* at Quarry Goluboy could not be inferred reliably due to near-fixation of the freshwater allele (Table 2). Whiskers correspond to values of *s* inferred from mean±st. dev. of allele frequency change.

Overall, estimated values of *s* were high: out of the 19 DIs, fifteen in Lake Ershovskoye, and twelve in Quarry Goluboy, had *s*>0.1 (Table 2, Figure 5). The selection coefficients estimated for each DI were not significantly correlated between the two freshwater populations (Figure 5; Spearman's rho = 0.30, p = 0.27), possibly due to the low number of DIs and high variance in estimation of *s* for individual DIs; however, the mean values of *s* inferred from Lake Ershovskoye (*s* = 0.16) and Quarry Goluboy (*s* = 0.13) were rather similar. In particular, DI IV-1, carrying the *EDA* allele, experienced *s* = 0.19 in Lake Ershovskoye, and *s* = 0.09 in Quarry Goluboy, consistent with the previously reported data on dynamics of armor phenotype over an even shorter time period [1]. The mean shift in allele frequency observed in Lake Ershovskoye was the largest in DI V-1, where freshwater allele frequency has changed from 0.1 to 0.56, corresponding to *s* = 0.255. This DI is centered on several nonsynonymous substitutions in gene *HTR3A*, a subunit of serotonin ligand-gated ion channel receptor with a wide spectrum of physiological functions. The close second was DI I-1 (*s_E_* = 0.247, *s_G_* = 0.212), a 470 kb long chromosomal inversion overlapping 27 genes, including the *ATP1A1* gene which encodes a well-studied Na+/K+ transporting ATPase; the differences between the freshwater and the marine allele of this gene include 6 amino acid substitutions, and may be responsible for the differences in osmoregulation between the two forms [56]. In Quarry Goluboy, the most radical change in allele frequency was observed at DI XII-2 (the freshwater allele has reached near-fixation here, so *s* is hard to estimate). This region overlaps the upstream region of the gene *OVGP1* (estrogen-dependent oviduct protein or mucin-9). This gene is involved in reproduction [57], and therefore is a likely target for positive selection; whether the strong selection associated with formation of freshwater phenotype is associated with arising reproductive isolation between the two forms should be the subject of a further study.

## Discussion

We have identified more than 18,000 marker SNPs that distinguish the two freshwater populations of *G. aculeatus* from the White Sea basin from the ancestral marine population. The great majority of these markers cluster within 19 short genomic regions, or DIs (Figure 2). All the DIs we found overlap with regions found in previous studies [15],[22], indicating a substantial degree of genetic parallelism in the origin of geographically distant freshwater populations and supporting the significance of the corresponding genomic regions for the process of adaptation.

Out of the 19 DIs, 12 overlap the regions reported both in [22] and [15] as responsible for adaptation to freshwater (Table 2), and the remaining 7 overlap loci reported in [15] only. Out of the 81 top-scoring regions described in [15], 71 regions are located within our DIs. 7 out of our 19 DIs (I-1, IV-1, IV-3, IV-4, VII-1, XIX-1, and XIX-2) overlap the top-scoring regions identified in [15] by both SOM/HMM and CSS analyses, and 12 more overlap the regions identified in [15] by less stringent criteria.

Extensive overlap between the adaptive regions described here for the White Sea populations and the ones reported from other regions confirms the previously described widespread parallelism in stickleback evolution [15],[22],[24],[27],[58]. A caveat is that both our study and the previous studies are specifically focused on finding genomic signatures of parallel evolution, and it is hard to infer the extent of parallelism from them directly. When only one, rather than two, marine-freshwater pairs of populations is used to identify marker SNPs, their number increases radically, likely due to addition of some true loci of population-specific positive selection, but also due to an increased number of false positives (Figure S3). Future studies involving many populations, and comparing loci involved in adaptation in some of the populations but not in others, are necessary to reveal the exact extent of parallelism.

The aggregated distribution of marker SNPs in the genome is probably determined by recombination patterns. For each DI, the observed increase of allele frequencies could be driven by positive selection favoring the “freshwater” allele at only one crucial polymorphic site, accompanied by hitch-hiking at surrounding neutral SNPs [59]. Alternatively, several distinct adaptive loci may be clustered together in regions of low recombination, with selection acting simultaneously at several such loci [15],[55]. Both these explanations are consistent with the fact that the three longest DIs found in our study all correspond to known inversions (DIs I-1, XI-1, XXI-1; Figure 2), i.e., the regions where recombination is minimal and hitch-hiking is most pronounced. In any case, our data do not imply that freshwater alleles at all the markers within a DI are favored by selection, even when a DI encompasses several protein-coding genes; the vast majority of the marker SNPs are probably neutral, and get fixed between the marine and freshwater populations due to hitch-hiking. Still, among the SNPs within the protein-coding genes, the nonsynonymous-to-synonymous ratio is higher for marker SNPs than for SNPs segregating within the ancestral marine population (Table 3), consistent with positive selection favoring marine-freshwater divergence at nonsynonymous sites [40]. Nine out of the 19 DIs do not contain any genes with nonsynonymous substitutions between freshwater and marine populations (Table 2), suggesting that evolution of regulatory mechanisms played a major role in the process of adaptation [15],[60],[61].

We observed a gradual increase of freshwater allele frequencies inside DIs (Figure 3 and Table S2) by exploring two young lake populations as well as two artificial populations established in 1978 from equal numbers of founder individuals of different forms. The selection coefficients estimated from the rates of increase of freshwater allele frequencies in Quarry Goluboy and Lake Ershovskoye are generally high (mean *s* = 0.13 and 0.16, respectively), implying rapid adaptation of *G. aculeatus* to the lacustrine environment (Table 2). The selection coefficient inferred for the *EDA* allele (*s* = 0.19 and 0.09) is somewhat lower than that inferred previously from short-term experimental populations (*s*∼0.5 [6]). Outside sticklebacks, the observed range of selection coefficients is comparable to that acting in the course of adaptation of *Biston betularia* peppered moth butterflies to predation (0.05–0.16 [2]), or estimated for the lactose-persistence allele in humans (0.014–0.19 [62]), and exceeds those estimated for *PDYN* promoter (<0.01 [63]) or genes involved in pigmentation (0.02–0.10, [64]) in humans. The variance of the frequencies of freshwater alleles among DIs is larger in the Quarry Malysh population than in the Quarry Goluboy population. The population of Malysh is much smaller than that of Goluboy and, therefore, this difference is possibly due to stronger genetic drift [65] in Malysh.

Within-DI densities of marker SNPs are rather high, and the marine and freshwater haplotypes differ from each other at over 1% of nucleotide sites within some DIs. Because most of marker SNPs are probably selectively neutral by themselves, this implies that these haplotypes diverged ∼10^6^ generations ago, assuming the mutation rate of 10^−8^ per nucleotide site per generation [66]. Such high divergence times are obviously inconsistent with *de novo* origin of freshwater alleles in each of the freshwater populations. Instead, they are consistent with repeated recruitment of the same ancient alleles in the course of establishment of different freshwater populations.

Only 5.0% of the marker SNPs identified under the strong marker criteria is located outside DIs. Some of these markers are in fact clustered, although they do not form DIs under our formal criteria. For example, at chromosome XIII, which carries no DIs, 11 out of the 18 markers are located within 0.5 Mb from each other. Notably, these marker SNPs also have the nonsynonymous-to-synonymous ratio substantially exceeding that in non-marker SNPs segregating within the marine population (Table 3). The fraction of nonsynonymous SNPs among coding marker SNPs is even higher outside DIs (17 out of 21) than within DIs (139 out of 285; Fisher's test, two-tailed P = 0.0056), suggesting that the fraction of marker SNPs under selection may be even higher outside than within DIs. Arguably, the differences in allelic frequencies of non-DI SNPs could be due to hitch-hiking with the DI marker SNPs; however, we see no difference in the rate of increase when the non-DI marker is located on a chromosome with a DI vs. without a DI (Figure S1A – natural populations; Figure S1B – artificial populations), and for markers at chromosomes with DIs, there is little correlation with the distance from the DI (Figure S2 – two natural lakes), suggesting that at least some of the non-island markers are also targets for selection. Plausibly, these SNPs are young and not yet surrounded by as many adjacent neutral hitchhikers accompanying them; the higher fraction of selected SNPs among them is therefore as expected. Still, each individual marker SNP located outside DIs is probably more likely to be indeed neutral, and to result from genetic drift and/or sampling bias, than a DI. Indeed, although the non-DI marker SNPs also increase their frequency in freshwater, this increase is less pronounced than within DIs (Figure 4), probably due to a higher fraction of neutral loci and/or weaker selection in the former.

7 out of the 19 detected DIs are “weak”. Analysis of such DIs reveals a number of patterns. In some of the weak DIs (VII-1 and IV-5, Figure 3A), the frequencies of freshwater alleles are close to 80%, but do not reach this threshold in either of the two populations from lakes Mashinnoye and Lobaneshskoye, so that the strong marker criteria do not hold. In others, although the frequencies of freshwater alleles seemingly increased gradually with the age of the population, they have not reached 80% in both lakes, perhaps due to weakness of selection favoring them (DIs II-1, IX-1, IX-2, and XI-1). In still others, freshwater alleles rapidly reach rather high frequencies in the young population, but these frequencies remain at the same level when the population's age increases (DI XXI-1, Figure 3A). The latter scenario could conceivably be due to the some form of balancing selection, perhaps due to interactions between the genes linked within the long inversion which constitutes DI XXI-1.

According to the “transporter hypothesis” [23], freshwater alleles are constantly present at low frequencies in the marine population, probably due to rare emigration from freshwater populations, and are recruited when a new freshwater population is established. The fact that alleles recruited in different freshwater populations tend to coincide, and that freshwater and marine haplotypes are highly divergent within DIs, support this hypothesis. Thus, a sort of balancing selection acts on the sites directly involved in adaptation to freshwater at the level of the global metapopulation [67] of *G. aculeatus*, keeping freshwater alleles from extinction. This metapopulation consists of the ancestral anadromous marine population and many derived residential freshwater populations. While the individual derived populations are often short-lived, the metapopulation has probably existed during much of the history of the species.

Although the ancestral alleles favored in the sea and the derived alleles favored in freshwater have coexisted for a long time, they have had only occasional opportunities to be separated by recombination from adjacent neutral polymorphisms. Indeed, this recombination can happen only in new residential populations, before fixation of alleles favored in freshwater together with linked neutral markers around them, or during the presumably short periods of time when such alleles exist in the sea before being recruited for formation of a new residential population. Thus, the width of a DI must be determined by the strength of selection favoring the freshwater alleles in freshwater populations and disfavoring them in the sea, by the recombination rate, and by the number of generations between the time when the freshwater allele has escaped from one freshwater population into the sea and the time when it has become recruited in another emerging lake residential population. Nucleotide diversity within strong DIs in our freshwater populations is somewhat lower than in the marine population, but not radically, indicating that adaptation to each freshwater lake has involved soft, rather than hard, selective sweeps [68]. Indeed, soft sweeps involve recruitment of multiple simultaneous sweeping haplotypes, and thus do not lead to a significant reduction in the nucleotide diversity around the selected site. Soft sweeps are likely when the sweeping alleles arise from pre-existing genetic variation rather than de novo mutations, and thus the lack of major reduction in diversity also supports the transporter hypothesis [23]. When a freshwater allele is brought into a new lake population by several individuals, nucleotide diversity is expected to increase on both sides of the DIs due to unequal length of freshwater DIs in founder individuals [69]; however, we see no such effect, probably due to the young age of our populations.

The very high evolutionary rate observed at several of the DIs during transition from marine to residential form could be attributed to pre-existing genomic regions, recruited from the standing variation of the marine population. Such “precast bricks” allow emerging freshwater population of sticklebacks to build rapidly a phenotype adapted to various challenges (salinity, parasites, energy metabolism, etc.) which it faces in the new environment. Plausibly, this form of evolution may be widespread beyond the stickleback model. Rapid emergence of parallel well-differentiated autochthonous flocks in the genus *Eubosmina* (Cladocera: Crustacea) in European lakes [70], flocks of *Labeobarbus* (Cyprinidae: Teleostei) in lakes and rivers of Ethiopia [71],[72], and genus *Salvelinus* (Salmonidae: Teleostei) [73],[74] could be a few out of many examples of this kind of evolution from precast bricks, during which new adaptive phenotypes are repeatedly created by rearrangement of ancient genetic elements, which were formed during earlier adaptive radiations and retained in ancestral population as standing variation.

## Materials and Methods

### Collection of samples and ethics statement

Fish were collected in June–August 2011 by scoop-net or landing-net, anaesthetized and euthanized with a tricaine methane sulphonate solution (MS222), and then immediately fixed in 96% ethanol on site. Fish euthanasia was conducted under the supervision of the Ethics Committee for Animal Research of the Koltzov Institute of Developmental Biology Russian Academy of Sciences. Location of lakes and quarries, estimated age of population, and sample size are presented in Table 1.

### Genome sequencing

Total genomic DNA was extracted from each individual using Wizard genomic DNA purification kit (Promega). Prior to library preparation, DNA samples of between 8 and 20 (Table 1) fish from the same population were pooled in equal proportions. Resulting pooled DNA samples were processed as described in the TruSeq DNA Sample Preparation Guide (Illumina). Library lengths were estimated using 2100 Bioanalyzer (Agilent). Libraries were quantified using fluorimetry with Qubit (Invitrogen) and real-time PCR (primers I-qPCR-1.1: AATGATACGGCGACCACCGAGAT and I-qPCR-2.1: CAAGCAGAAGACGGCATACGA) and diluted to final concentration of 9 pM. Diluted libraries were clustered on a paired-end flow cell using cBot instrument and sequenced using HiSeq2000 sequencer with TruSeq SBS Kit v3-HS (Illumina), read length 101 from each end. Sequences for each population are available at the NCBI Short Read Archive (http://www.ncbi.nlm.nih.gov/Traces/sra; accession number of the project SRP023197).

### Genome mapping and sequence analysis

The reads were mapped onto the reference genome of *G. aculeatus* downloaded from the UCSC (http://genome.ucsc.edu/) using bwa aln program of the BWA (Burrows-Wheeler Alignment Tool) package (http://bio-bwa.sourceforge.net/). Output was then converted to SAM format using bwa sampe. Next, data were processed with picard (http://picard.sourceforge.net/) in order to remove duplicated reads. We identified SNPs in all populations using program mpileup of the samtools package (http://samtools.sourceforge.net/). For SNP calling, different depth cutoffs were used for different populations due to differences in read coverage among populations: >10 for Nilma, Malysh, Goluboy and residential fish from Ershovskoye, and >5 for Mashinnoye, Lobaneshskoye, Martsy, and anadromous fish from Ershovskoye. To minimize sequencing errors, positions with base qualities lower than 40 within a population were excluded from the analysis. As an alternative approach to SNP calling, we used UnifiedGenotyper program from GATK package (http://www.broadinstitute.org/gatk/) with the same coverage cutoffs. This led to a larger pool of marker SNPs, but similar clustering patterns: all the DIs identified with the program mpileup were observed, as well as several new clusters (Table S5). The patterns of allele frequency dynamics were qualitatively similar under mpileup and GATK SNP calling (Table S6). Overall, a higher proportion of marker SNP were located outside DIs using GATK (Table S5); therefore, we chose to use mpileup for the results in the main text.

Positions of genes were derived from Ensembl database release 72 (http://www.ensembl.org/)

### Clustering of SNPs along the genome

To define DIs, we merged any two regions with above-threshold numbers of marker SNPs that were closer than 40 Kb to each other. Generally, this merging procedure described our data well: for example, it prevented splitting several DIs all corresponding to a single known inversion, or division of one DI into several due to the lack of coverage in some regions. Not merging adjacent DIs led to a drastic increase in their number [71]; the qualitative patterns of allele dynamics remain the same (Table S7). This is as expected, because recombination (average recombination rate in threespine stickleback is 3.11 cM/Mb [39]) is not likely to occur between regions located so close to each other (less than 40 Kb) over the considered timescales.

### Allele frequencies validation

To validate our estimates of allelic frequencies based on high-throughput sequencing data, we also genotyped each fish used for pooled DNA sample with an allele-specific set of primers for markers located within several of the DIs. For this purpose, we designed 8 allele-specific sets of primers for 7 of the DIs (one DI was genotyped with two sets of primers). Each set consisted of three primers: two allele-specific, and one anchor primer. Additionally, we used previously published primers Stn382 [27] to genotype DI IV-1. Primers, positions of target SNPs, and PCR annealing temperature for each pair of primers are presented in Table S3. Two allele-specific PCR reactions (each with one allele-specific and one common anchor primers) were set for each individual, and the second PCR product was applied in the same well of agarose gel as the first PCR reaction after 5 min of running the gel. Individuals with one or both PCR products were categorized as homo- or heterozygotes, respectively. The obtained allele frequencies matched well those estimated from high-throughput sequencing data (Table S4).

### Age of freshwater populations

As a proxy for the time of formation of natural residential populations, we use the time of desalination. Before complete desalination, a lake is meromictic, and contains two water layers – a higher freshwater layer, and a lower saltwater layer, forming a halocline. This halocline prevents proper oxygenation; as a result, the lake becomes anoxic every winter, causing extirpation of residential populations [75].

### Estimation of selection coefficients

For each DI, we calculated the average frequency of a freshwater allele over all marker SNPs (Table S2). We estimated generation time using length-cohort analysis, which revealed two cohorts present in each lake: immature one-year old fish, and a second-year class which participated in reproduction. Presence of three year-old and older fish in the lake population was negligible. Therefore, we assumed generation length of two years; reproduction at older ages will lead to underestimation of *s*. The ages of Goluboy and Malysh populations are known to be 34 years (17 generations). The Ershovskoye freshwater population is known to be 34 years old or younger [33]; we assumed its age to be 34 years (17 generations), and younger age will again lead to underestimation of the true *s*.

The initial frequencies of freshwater alleles in the two artificial populations, Goluboy and Malysh, were assumed to equal 0.5. In 1978, all fish in Lake Ershovskoye had phenotypic composition similar to that of a typical marine population [33]. We assume that the initial allelic frequencies in the Lake Ershovskoye matched the frequencies in marine populations, i.e., *p*_0_∼0.1. Selection coefficient *s* was calculated from the per generation change in allele frequency under the assumption that this change is driven by selection alone (Eqn. 3.2 in [76], assuming h = 0.5).

## Supporting Information

Figure S1 Mean frequencies of freshwater alleles at marker SNPs obtained under the strong criterion and located outside identified DIs, at freshwater populations of different ages. (**A**) Natural populations. The horizontal axis shows the approximate ages of populations, ranging (left to right) from two marine populations (∼0 years) to recent (∼34 and ∼250 years) and older lake populations (∼600 and ∼700 years). Whiskers, standard deviation. (**B**) Experimental populations. For each of the DIs of marker SNPs, the assumed initial 50% frequency of freshwater alleles (black line) and their current frequencies are shown for two experimental populations: quarries Malysh (left) and Goluboy (right), each started in 1978. Dashes, boxes and whiskers correspond to the median, standard deviation, and 5^th^ and 95^th^ percentiles, respectively.
(EPS)

Figure S2 Mean frequencies of freshwater alleles at marker SNPs obtained under the strong criterion and located outside identified DIs depending on the distance from the DI. The horizontal axis shows, in logarithmic scale, the distance of the marker SNP from the nearest DI in bp and the y axis shows the frequency of freshwater allele in marker SNP.
(EPS)

Figure S3 Comparison of mean frequencies of freshwater alleles at marker SNPs located within and outside of DIs defined by only one marine-freshwater population pair, at freshwater populations of different ages. Each two boxes correspond to the four young freshwater populations (anadromous from Lake Ershovskoye, Lake Martsy, Quarry Malysh, Quarry Goluboy). Dashes, boxes and whiskers correspond to the median, standard deviation, and 5^th^ and 95^th^ percentiles, respectively. Red, marker SNPs (under the strong criterion) located within identified DIs; purple, marker SNPs (under the strong criterion) located outside DIs.
(TIF)

Table S1 Genes overlapping the 19 DIs of marker SNPs, their functions, and types of substitutions associated with them.
(XLSX)

Table S2 Average frequencies of freshwater alleles in each population for each of the 19 DIs found in our study, mean ± st. dev. Asterisks denote the DIs identified under the weak criteria.
(PDF)

Table S3 List of allele-specific primers used for DIs validation.
(PDF)

Table S4 Comparison of freshwater allele frequencies based on allele-specific PCR and on calculation of marker SNP frequencies.
(PDF)

Table S5 Comparison of the two programs mpileup and GATK for identification marker SNPs. Bold font denotes DIs identified under the strong criteria, non-bold font denotes DIs identified under the weak criteria.
(PDF)

Table S6 Average frequencies of freshwater alleles in each population for each of the 35 DIs defined with the GATK in our study, mean ± st. dev.
(PDF)

Table S7 Mean frequencies of freshwater alleles in each population for DIs found in our study without merging procedure, mean ± st. dev.
(PDF)
