# Supplementary figures and images for: Fast Evolution from Precast Bricks: Genomics of Young Freshwater Populations of Threespine Stickleback Gasterosteus aculeatus
Source: PLoS Genet. 2014 Oct 9;10(10):e1004696. doi: 10.1371/journal.pgen.1004696 (PMC4191950; doi:10.1371/journal.pgen.1004696)

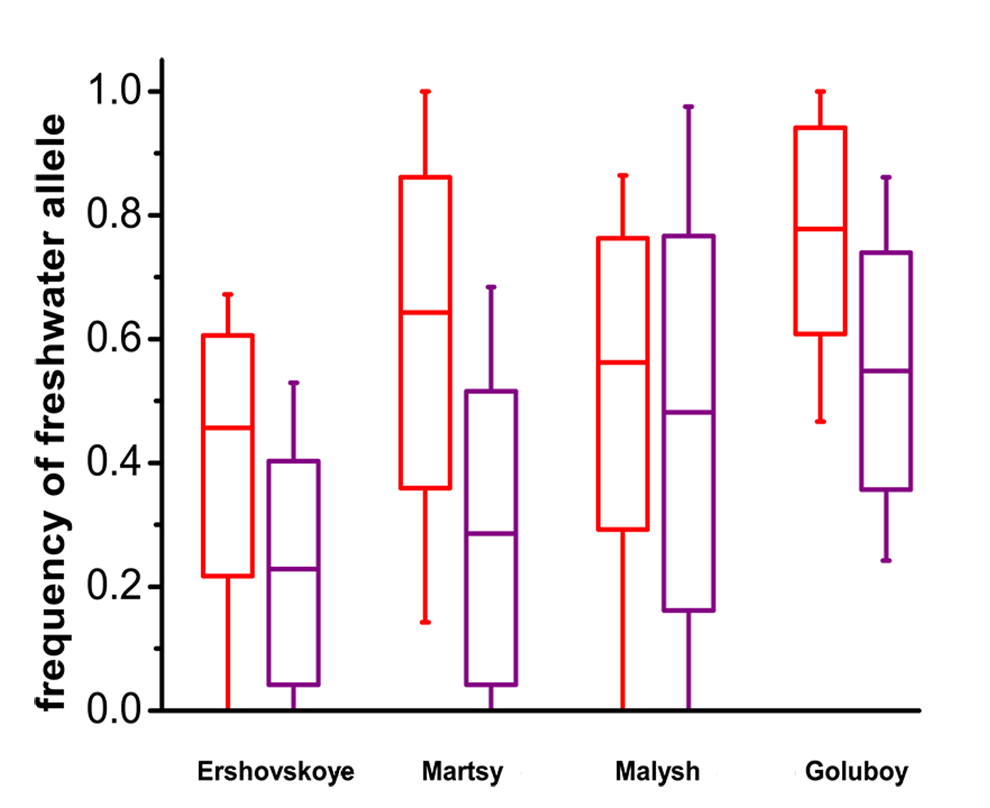

Supplement: Figure S3 — Comparison of mean frequencies of freshwater alleles at marker SNPs located within and outside of DIs defined by only one marine-freshwater population pair, at freshwater populations of different ages. Each two boxes correspond to the four young freshwater populations (anadromous from Lake Ershovskoye, Lake Martsy, Quarry Malysh, Quarry Goluboy). Dashes, boxes and whiskers correspond to the median, standard deviation, and 5th and 95th percentiles, respectively. Red, marker SNPs (under the strong criterion) located within identified DIs; purple, marker SNPs (under the strong criterion) located outside DIs. (TIF) [file pgen.1004696.s003.tif]
