# Supplementary material for: Fast Evolution from Precast Bricks: Genomics of Young Freshwater Populations of Threespine Stickleback Gasterosteus aculeatus
Source: PLoS Genet. 2014 Oct 9;10(10):e1004696. doi: 10.1371/journal.pgen.1004696 (PMC4191950; doi:10.1371/journal.pgen.1004696)
Supplement: Table S2 — Average frequencies of freshwater alleles in each population for each of the 19 DIs found in our study, mean ± st. dev. Asterisks denote the DIs identified under the weak criteria. (PDF) [file pgen.1004696.s005.pdf]

DI	Start	End	Nilma	Ershovskoye (anadromous)	Ershovskoye (residential)	Martsy	Goluboy	Malysh	Lobaneshskoye	Mashinnoye
I-1	21,487,998	21,960,119	0.07±0.05	0.06±0.06	0.54±0.09	0.77±0.15	0.87±0.11	0.56±0.09	1±0.01	1±0.01
II-1*	14,874,366	14,898,826	0.1±0.06	0.05±0.06	0.23±0.04	0.27±0.11	0.78±0.06	0.59±0.05	0.71±0.08	0.66±0.1
IV-1	12,803,780	12,881,296	0.12±0.06	0.07±0.07	0.38±0.09	0.67±0.12	0.69±0.09	0.5±0.09	1±0.02	0.99±0.03
IV-2	13,930,002	13,959,331	0.12±0.05	0.1±0.06	0.46±0.09	0.67±0.11	0.71±0.06	0.52±0.08	1±0.01	1±0
IV-3	19,811,922	19,914,666	0.14±0.05	0.12±0.06	0.44±0.1	0.68±0.12	0.8±0.12	0.61±0.1	1±0.02	1±0.02
IV-4	23,954,634	23,981,981	0.15±0.04	0.08±0.07	0.48±0.08	0.73±0.11	0.7±0.07	0.67±0.05	1±0	0.99±0.05
IV-5*	26,016,955	26,166,536	0.14±0.04	0.11±0.06	0.4±0.1	0.71±0.13	0.57±0.09	0.67±0.07	1±0.03	0.69±0.12
V-1	2,482,209	2,501,295	0.11±0.05	0.13±0.06	0.56±0.1	0.73±0.13	0.89±0.11	1±0	1±0.02	1±0
VII-1*	17,982,351	18,002,671	0.08±0.04	0.05±0.06	0.05±0.05	0.23±0.11	0.92±0.11	0.83±0.08	0.58±0.07	1±0
IX-1*	8,521,935	8,537,559	0.11±0.05	0.04±0.06	0.16±0.07	0.43±0.11	0.55±0.09	0.69±0.06	0.55±0.04	0.79±0.15
IX-2*	8,901,816	8,910,115	0.13±0.04	0.08±0.07	0.22±0.09	0.46±0.12	0.55±0.09	0.16±0.28	0.63±0.15	0.72±0.2
IX-3	9,208,158	9,227,809	0.14±0.06	0.05±0.06	0.41±0.09	0.66±0.09	0.75±0.1	0.67±0.06	0.97±0.05	1±0
IX-4	10,334,101	10,353,801	0.12±0.05	0.07±0.06	0.28±0.08	0.73±0.09	0.7±0.07	0.9±0.08	1±0.01	1±0.01
XI-1*	5,445,757	5,855,124	0.05±0.05	0.03±0.05	0.15±0.09	0.48±0.12	0.78±0.1	0.1±0.08	0.73±0.11	0.79±0.14
XII-1	14,338,229	14,358,336	0.15±0.04	0.1±0.06	0.12±0.06	0.41±0.11	0.84±0.11	0.83±0.08	1±0.02	1±0
XII-2	16,522,028	16,538,810	0.08±0.06	0.05±0.07	0.32±0.07	0.41±0.1	0.98±0.03	0.29±0.06	0.94±0.08	0.99±0.02

XIX-1	2,449,903	2,581,858	0.13±0.05	0.07±0.06	0.36±0.1	0.92±0.13	0.75±0.12	0.68±0.09	1±0.02	1±0.01
XIX-2	14,787,904	14,799,088	0.09±0.06	0.05±0.06	0.24±0.07	0.49±0.18	0.61±0.12	0.37±0.13	0.99±0.04	0.98±0.05
XXI-1*	5,759,879	7,486,635	0.01±0.02	0.03±0.05	0.36±0.09	0.59±0.13	0.47±0.1	0.01±0.05	0.69±0.11	0.59±0.08

Table S2. Average frequencies of freshwater alleles in each population for each of the 19 DIs found in our study, mean ± st. dev. Asterisks denote the DIs identified under the weak criteria.
