# Supplementary material for: Fast Evolution from Precast Bricks: Genomics of Young Freshwater Populations of Threespine Stickleback Gasterosteus aculeatus
Source: PLoS Genet. 2014 Oct 9;10(10):e1004696. doi: 10.1371/journal.pgen.1004696 (PMC4191950; doi:10.1371/journal.pgen.1004696)
Supplement: Table S3 — List of allele-specific primers used for DIs validation. (PDF) [file pgen.1004696.s006.pdf]

**DI/locus**	**SNP position**	**Primer**	**Primer sequence**	**Used with**	**Annealing T (°C)**
I-1	chrI:21,698,392	Chr1_F_R	AGTCACTTACAAAACCAAGAAGCTTTC	Chr1_FS_F	68
		Chr1_S_R	TCAACTTACAAAACCAAGAAGCGG	Chr1_FS_F	65
		Chr1_FS_F	AGGCCTTTTCACAGGCAGCCTC		
IV-1	chrIV:12,802,776	Stn382F (Ref. 27)	CCCTTAGAGAATTTCCTAGCAG	Stn382R	56
	- chrIV:12,802,926	Stn382R (Ref. 27)	CTTGTCCCGGATCATACGC		
IV-3	chrIV:19,875,745	Chr4-3_F_F	TATTAAGCACTGATCCAATTCTTAT	Chr4-3_FS_R	65
		Chr4-3_S_F	TATTAAGCACTGATCCAATTCTTGA	Chr4-3_FS_R	65
		Chr4-3_FS_R	CTCTCAGCAAACTGAGCACTGCAGT		
IV-4	chrIV:23,963,712	Chr4-4_F_R	ATCCTGGTTCTGTGACCTAATGCTC	Chr4-4_FS_F	63
		Chr4-4_S_R	ATCCTGGTTCTGTGTCCTAATGTTG	Chr4-4_FS_F	63
		Chr4-4_FS_F	AAGTGGCGTGGCACAGAGAAGTAC		
IX-4	chrIX:10,338,733	Chr9-1_F_F	AGCGCTTCCAAAGGAGTTCTGAG	Chr9-1_SF_R	68
		Chr9-1_S_F	CAGCGCTTCCAAAGGAGTTCTGTA	Chr9-1_SF_R	63
		Chr9-1_SF_R	TGTGCCAACCCGCCATCATA		
XIX-1a	chrXIX:2,475,523	Chr19a_F_F	GGCTACAACAACAAATTCATGCACT	Chr19a_SF_R	65
		Chr19a_S_F	GACTACAACAACAACTTCATGCATA	Chr19a_SF_R	65
		Chr19a_SF_R	ATGAGCGACAACAATAAACAAATGC		

XIX-1b	chrXIX:2,460,316	Chr19b_F_F	AGACAAACTGCCACAAAAGATGAAACA	Chr19b_SF_R	68
		Chr19b_S_F	AGACAAACTGCCACAAAAAATGAAATG	Chr19b_SF_R	63
		Chr19b_SF_R	CTCTGTAAACGATTGGTCCAGTGTCAC		
XXI-1*	chrXXI:6,926,742	Chr21b_F_F	CAATGATTTAACCTGTTGACAGC	Chr21b_FS_R	68
		Chr21b_S_F	GTCAATGATTTAACCTGTTGACATA	Chr21b_FS_R	63
		Chr21b_FS_R	CAGTAATGGTGGGTCAGTGTACGT		

Table S3. List of allele-specific primers used for DIs validation.
