# Supplementary material for: Fast Evolution from Precast Bricks: Genomics of Young Freshwater Populations of Threespine Stickleback Gasterosteus aculeatus
Source: PLoS Genet. 2014 Oct 9;10(10):e1004696. doi: 10.1371/journal.pgen.1004696 (PMC4191950; doi:10.1371/journal.pgen.1004696)
Supplement: Table S4 — Comparison of freshwater allele frequencies based on allele-specific PCR and on calculation of marker SNP frequencies. (PDF) [file pgen.1004696.s007.pdf]

| DI/locus | Nilma | | Ershovskoye (anadromous) | | Ershovskoye (residential) | | Martsy | |
|---|---|---|---|---|---|---|---|---|
| | **PCR** | **Illumina** | **PCR** | **Illumina** | **PCR** | **Illumina** | **PCR** | **Illumina** |
| I-1 | 0.125 | 0.07±0.05 | 0 | 0.06±0.06 | 0.500 | 0.54±0.09 | 0.750 | 0.77±0.15 |
| IV-1 | 0.125 | 0.12±0.06 | 0.125 | 0.07±0.07 | 0.417 | 0.38±0.09 | 0.700 | 0.67±0.12 |
| IV-3 | 0.156 | 0.14±0.05 | 0.15 | 0.12±0.06 | 0.458 | 0.44±0.1 | 0.750 | 0.68±0.12 |
| IV-4 | 0.156 | 0.15±0.04 | 0.2 | 0.08±0.07 | 0.458 | 0.48±0.08 | 0.650 | 0.73±0.11 |
| IX-4 | 0.156 | 0.12±0.05 | 0.05 | 0.07±0.06 | 0.208 | 0.28±0.08 | 0.700 | 0.73±0.09 |
| XIX-1a | 0.094 | 0.13±0.05 | - | 0.07±0.06 | 0.375 | 0.36±0.1 | 0.750 | 0.92±0.13 |
| XIX-1b | 0.094 | 0.13±0.05 | 0.05 | 0.07±0.06 | 0.250 | 0.36±0.1 | 0.850 | 0.92±0.13 |
| XXI-1* | 0.000 | 0.01±0.02 | - | 0.03±0.05 | 0.333 | 0.36±0.09 | 0.550 | 0.59±0.13 |

| DI/locus | Goluboy | | Malysh | | Lobaneshskoye | | Mashinnoye | |
|---|---|---|---|---|---|---|---|---|
| | **PCR** | **Illumina** | **PCR** | **Illumina** | **PCR** | **Illumina** | **PCR** | **Illumina** |
| I-1 | 0.850 | 0.87±0.11 | 0.525 | 0.56±0.09 | 1.000 | 1±0.01 | 1.000 | 1±0.01 |
| IV-1 | 0.700 | 0.69±0.09 | 0.525 | 0.5±0.09 | 0.938 | 1±0.02 | 0.900 | 0.99±0.03 |

| | | | | | | | | |
|---|---|---|---|---|---|---|---|---|
| IV-3 | 0.875 | 0.8±0.12 | 0.650 | 0.61±0.1 | 1.000 | 1±0.02 | 1.000 | 1±0.02 |
| IV-4 | 0.700 | 0.7±0.07 | 0.675 | 0.67±0.05 | 1.000 | 1±0 | 0.900 | 0.99±0.05 |
| IX-4 | 0.750 | 0.7±0.07 | 0.700 | 0.9±0.08 | 1.000 | 1±0.01 | 1.000 | 1±0.01 |
| XIX-1a | 0.775 | 0.75±0.12 | 0.650 | 0.68±0.09 | 1.000 | 1±0.02 | 1.000 | 1±0.01 |
| XIX-1b | 0.775 | 0.75±0.12 | 0.675 | 0.68±0.09 | 1.000 | 1±0.02 | 1.000 | 1±0.01 |
| XXI-1* | 0.350 | 0.47±0.1 | 0.000 | 0.01±0.05 | 0.688 | 0.69±0.11 | 0.450 | 0.59±0.08 |

**Table S4. Comparison of freshwater allele frequencies based on allele-specific PCR and on calculation of marker SNP frequencies.**
