# Supplementary material for: Fast Evolution from Precast Bricks: Genomics of Young Freshwater Populations of Threespine Stickleback Gasterosteus aculeatus
Source: PLoS Genet. 2014 Oct 9;10(10):e1004696. doi: 10.1371/journal.pgen.1004696 (PMC4191950; doi:10.1371/journal.pgen.1004696)
Supplement: Table S5 — Comparison of the two programs mpileup and GATK for identification marker SNPs. Bold font denotes DIs identified under the strong criteria, non-bold font denotes DIs identified under the weak criteria. (PDF) [file pgen.1004696.s008.pdf]

	**mpileup**				**GATK**			
**#chr**	**start**	**end**	**#markers**	**length**	**start**	**end**	**#markers**	**length**
chrI					12,386,698	12,398,661	30	11,964
chrI	**21,487,998**	**21,960,119**	**4,186**	**472,122**	**21,486,723**	**21,960,546**	**4,071**	**473,824**
chrII					**400,967**	**410,723**	**11**	**9,757**
chrII					**6,125,444**	**6,133,126**	**11**	**7,683**
chrII	14,874,366	14,898,826	73	24,461	14,868,709	14,903,504	133	34,796
chrII					15,917,413	15,927,588	22	10,176
chrIII					**12,393,053**	**12,401,196**	**12**	**8,144**
chrIV	**12,803,780**	**12,881,296**	**285**	**77,517**	**12,801,904**	**12,884,281**	**368**	**82,378**
chrIV	**13,930,002**	**13,959,331**	**168**	**29,330**	**13,930,002**	**13,965,650**	**180**	**35,649**
chrIV	**19,811,922**	**19,914,666**	**209**	**102,745**	**19,811,668**	**19,914,285**	**235**	**102,618**
chrIV					**21,119,742**	**21,129,459**	**13**	**9,718**
chrIV	**23,954,634**	**23,981,981**	**48**	**27,348**	**23,926,875**	**23,982,004**	**126**	**55,130**
chrIV	26,016,955	26,166,536	252	149,582	26,014,651	26,173,878	364	159,228
chrIV					**27,506,950**	**27,529,635**	**24**	**22,686**
chrV	**2,482,209**	**2,501,295**	**65**	**19,087**	**2,482,368**	**2,507,363**	**52**	**24,996**
chrVII					**4,673,859**	**4,684,431**	**11**	**10,573**
chrVII	17,982,351	18,002,671	84	20,321	17,971,522	18,009,091	131	37,570
chrVIII					8,251,224	8,259,722	20	8,499
chrIX	8,521,935	8,537,559	44	15,625	8,511,171	8,537,543	106	26,373

chrIX	8,901,816	8,910,115	20	8,300	8,901,816	8,910,874	42	9,059
chrIX	**9,208,158**	**9,227,809**	**46**	**19,652**	**9,129,464**	**9,232,163**	**109**	**102,700**
chrIX	**10,334,101**	**10,353,801**	**114**	**19,701**	**10,327,579**	**10,353,801**	**90**	**26,223**
chrIX					12,066,123	12,181,815	67	115,693
chrX					8,301,947	8,311,946	20	10,000
chrXI	5,445,757	5,855,124	1,237	409,368	5,436,982	5,915,286	3,112	478,305
chrXII	**14,338,229**	**14,358,336**	**91**	**20,108**	**14,329,844**	**14,357,195**	**75**	**27,352**
chrXII	**16,522,028**	**16,538,810**	**24**	**16,783**	**16,521,493**	**16,538,557**	**30**	**17,065**
chrXIII					8,447,744	8,457,144	29	9,401
chrXIV					**11,349,733**	**11,361,406**	**21**	**11,674**
chrXVIII					**882,850**	**906,041**	**63**	**23,192**
chrXIX	**2,449,903**	**2,581,858**	**277**	**131,956**	**2,449,903**	**2,575,655**	**252**	**125,753**
chrXIX					**12,488,785**	**12,497,245**	**10**	**8,461**
chrXIX	**14,787,904**	**14,799,088**	**21**	**11,185**	14,775,739	14,807,299	98	31,561
chrXX					8,626,815	8,651,960	50	25,146
chrXXI	5,759,879	7,486,635	6,900	1,726,757	5,757,848	7,491,073	13,508	1,733,226
Total			14,144	3,301,948			23,496	3,886,573

Table S5. Comparison of the two programs mpileup and GATK for identification marker SNPs. Bold font denotes DIs identified under the strong criteria, non-bold font denotes DIs identified under the weak criteria.
