# Supplementary material for: Fast Evolution from Precast Bricks: Genomics of Young Freshwater Populations of Threespine Stickleback Gasterosteus aculeatus
Source: PLoS Genet. 2014 Oct 9;10(10):e1004696. doi: 10.1371/journal.pgen.1004696 (PMC4191950; doi:10.1371/journal.pgen.1004696)
Supplement: Table S6 — Average frequencies of freshwater alleles in each population for each of the 35 DIs defined with the GATK in our study, mean ± st. dev. (PDF) [file pgen.1004696.s009.pdf]

DI	Start	End	Nilma	Ershovskoye (anadromous)	Ershovskoye (residential)	Martsy	Goluboy	Malysh	Lobaneshskoye	Mashinnoye
I-1*	12,386,698	12,398,661	0.14±0.04	0.04±0.06	0.16±0.11	0.46±0.17	0.73±0.08	0.22±0.21	0.74±0.11	0.95±0.09
I-2	21,486,723	21,960,546	0.09±0.05	0.07±0.07	0.54±0.1	0.79±0.11	0.85±0.08	0.58±0.11	0.99±0.03	1±0.02
II-1	400,967	410,723	0.17±0.02	0.03±0.04	0.23±0.08	0.45±0.16	0.71±0.07	0.43±0.05	0.83±0.03	0.99±0.03
II-2	6,125,444	6,133,126	0.15±0.04	0.12±0.08	0.53±0.12	0.74±0.11	0.84±0.06	0.48±0.2	0.97±0.06	1±0
II-3*	14,868,709	14,903,504	0.11±0.04	0.06±0.07	0.26±0.08	0.29±0.12	0.79±0.08	0.6±0.07	0.73±0.1	0.69±0.1
II-4*	15,917,413	15,927,588	0.13±0.05	0.04±0.06	0.43±0.13	0.43±0.14	0.69±0.12	0.63±0.12	0.63±0.13	0.75±0.1
III-1	12,393,053	12,401,196	0.14±0.03	0.06±0.07	0.43±0.14	0.52±0.13	0.74±0.1	0.88±0.04	0.94±0.07	0.98±0.05
IV-1	12,801,904	12,884,281	0.13±0.04	0.06±0.07	0.37±0.1	0.69±0.13	0.69±0.1	0.5±0.1	0.98±0.04	0.97±0.06
IV-2	13,930,002	13,965,650	0.12±0.05	0.1±0.07	0.46±0.1	0.74±0.11	0.73±0.08	0.54±0.1	1±0.02	1±0.02
IV-3	19,811,668	19,914,285	0.14±0.04	0.12±0.06	0.44±0.12	0.73±0.12	0.82±0.09	0.63±0.11	0.98±0.04	0.98±0.05
IV-4	21,119,742	21,129,459	0.15±0.04	0.13±0.05	0.38±0.1	0.71±0.19	0.79±0.12	0.91±0.07	1±0.02	0.98±0.06
IV-5	23,926,875	23,982,004	0.15±0.04	0.07±0.07	0.46±0.12	0.76±0.1	0.71±0.09	0.7±0.08	1±0.02	0.87±0.06
IV-6*	26,014,651	26,173,878	0.14±0.04	0.12±0.07	0.41±0.09	0.78±0.1	0.61±0.09	0.69±0.08	1±0.02	0.74±0.1
IV-7	27,506,950	27,529,635	0.11±0.05	0.18±0.02	0.19±0.07	0.42±0.14	0.74±0.13	0±0.01	0.98±0.05	0.96±0.06
V-1	2,482,368	2,507,363	0.11±0.05	0.11±0.08	0.56±0.1	0.79±0.12	0.86±0.06	1±0.01	0.96±0.05	1±0.01
VII-1	4,673,859	4,684,431	0.07±0.05	0.12±0.09	0.2±0.1	0.31±0.18	0.76±0.07	0.41±0.18	0.92±0.08	1±0.02

VII-2*	17,971,522	18,009,091	0.09±0.04	0.07±0.07	0.06±0.06	0.24±0.11	0.88±0.06	0.84±0.05	0.58±0.09	1±0.02
VIII-1*	8,251,224	8,259,722	0.12±0.03	0.04±0.07	0.11±0.05	0.28±0.13	0.45±0.1	0.3±0.14	0.58±0.11	0.81±0.16
IX-1*	8,511,171	8,537,543	0.12±0.04	0.05±0.06	0.15±0.06	0.46±0.14	0.57±0.09	0.7±0.06	0.58±0.09	0.78±0.12
IX-2*	8,901,816	8,910,874	0.14±0.04	0.07±0.08	0.19±0.1	0.47±0.15	0.54±0.13	0.28±0.33	0.68±0.17	0.78±0.19
IX-3	9,129,464	9,232,163	0.13±0.04	0.06±0.07	0.42±0.09	0.63±0.13	0.71±0.12	0.65±0.07	0.89±0.06	0.97±0.06
IX-4	10,327,579	10,353,801	0.13±0.04	0.08±0.07	0.29±0.09	0.79±0.08	0.71±0.08	0.88±0.04	0.98±0.05	1±0
IX-5	12,066,123	12,181,815	0.1±0.06	0.09±0.07	0.38±0.13	0.54±0.16	0.55±0.16	0.65±0.21	0.7±0.09	0.73±0.15
X-1	8,301,947	8,311,946	0.07±0.06	0.03±0.06	0.23±0.07	0.22±0.1	0.66±0.07	0.7±0.07	0.61±0.1	0.73±0.09
XI-1	5,436,982	5,915,286	0.08±0.05	0.05±0.06	0.16±0.07	0.52±0.14	0.81±0.08	0.13±0.07	0.76±0.1	0.8±0.1
XII-1	14,329,844	14,357,195	0.15±0.04	0.11±0.07	0.13±0.07	0.41±0.13	0.85±0.07	0.83±0.06	0.98±0.05	1±0.02
XII-2	16,521,493	16,538,557	0.1±0.04	0.09±0.08	0.32±0.09	0.4±0.13	0.94±0.04	0.28±0.07	0.88±0.05	1±0.02
XIII-1	8,447,744	8,457,144	0.13±0.05	0.07±0.07	0.15±0.04	0.81±0.1	0.73±0.08	0.59±0.17	0.86±0.03	0.99±0.03
XIV-1	11,349,733	11,361,406	0.11±0.06	0.15±0.05	0.3±0.06	0.18±0.09	0.73±0.08	0.27±0.06	0.87±0.05	0.89±0.08
XVIII-1*	882,850	906,041	0.13±0.04	0.09±0.07	0.22±0.08	0.48±0.13	0.29±0.12	0.72±0.12	0.78±0.09	0.68±0.12
XIX-1	2,449,903	2,575,655	0.14±0.05	0.07±0.07	0.37±0.1	0.83±0.1	0.78±0.11	0.71±0.1	0.98±0.05	0.98±0.05
XIX-2	12,488,785	12,497,245	0.1±0.04	0.02±0.04	0.01±0.04	0.09±0.1	0.5±0.08	0.41±0.1	0.98±0.06	0.98±0.05
XIX-3*	14,775,739	14,807,299	0.09±0.05	0.07±0.07	0.21±0.09	0.48±0.16	0.58±0.16	0.32±0.11	0.84±0.15	0.8±0.15

XX-1*	8,626,815	8,651,960	0.11±0.04	0.16±0.05	0.43±0.1	0.65±0.13	0.7±0.11	0.54±0.14	0.87±0.15	0.72±0.13
XXI-1*	5,757,848	7,491,073	0.01±0.02	0.04±0.06	0.38±0.11	0.64±0.13	0.51±0.11	0.01±0.06	0.73±0.1	0.62±0.1

Table S6. Average frequencies of freshwater alleles in each population for each of the 35 DIs defined with the GATK in our study, mean ± st. dev.
