# Supplementary material for: Fast Evolution from Precast Bricks: Genomics of Young Freshwater Populations of Threespine Stickleback Gasterosteus aculeatus
Source: PLoS Genet. 2014 Oct 9;10(10):e1004696. doi: 10.1371/journal.pgen.1004696 (PMC4191950; doi:10.1371/journal.pgen.1004696)
Supplement: Table S7 — Mean frequencies of freshwater alleles in each population for DIs found in our study without merging procedure, mean ± st. dev. (PDF) [file pgen.1004696.s010.pdf]

DI	Start	End	Nilma	Ershovskoye (anadromous)	Ershovskoye (residential)	Martsy	Goluboy	Malysh	Lobaneshskoye	Mashinnoye
chrI	21,487,998	21,523,284	0.07±0.05	0.06±0.06	0.52±0.08	0.74±0.13	0.85±0.12	0.55±0.08	1±0.01	1±0
chrI	21,524,624	21,549,574	0.06±0.06	0.06±0.07	0.58±0.09	0.8±0.15	0.88±0.11	0.58±0.11	1±0.02	1±0.02
chrI	21,553,963	21,871,117	0.07±0.05	0.06±0.06	0.54±0.09	0.77±0.15	0.87±0.11	0.57±0.09	1±0.01	1±0.01
chrI	21,872,973	21,960,119	0.07±0.05	0.06±0.06	0.53±0.09	0.77±0.15	0.86±0.12	0.56±0.1	1±0.02	1±0.02
chrII*	14,874,366	14,898,826	0.1±0.06	0.05±0.06	0.23±0.04	0.27±0.11	0.78±0.06	0.59±0.05	0.71±0.08	0.66±0.1
chrIV	12,803,780	12,843,813	0.13±0.06	0.07±0.07	0.41±0.09	0.69±0.1	0.69±0.08	0.49±0.09	1±0.02	0.99±0.03
chrIV	12,859,609	12,881,296	0.12±0.05	0.08±0.06	0.33±0.08	0.62±0.13	0.68±0.09	0.5±0.09	0.99±0.03	0.99±0.02
chrIV	13,930,002	13,959,331	0.12±0.05	0.1±0.06	0.46±0.09	0.67±0.11	0.71±0.06	0.52±0.08	1±0.01	1±0
chrIV	19,811,922	19,821,885	0.09±0.07	0.06±0.08	0.32±0.09	0.62±0.07	0.74±0.08	0.55±0.16	0.97±0.05	0.96±0.05
chrIV	19,851,933	19,914,666	0.14±0.04	0.12±0.06	0.45±0.09	0.69±0.12	0.81±0.12	0.62±0.09	1±0	1±0
chrIV	23,954,634	23,981,981	0.15±0.04	0.08±0.07	0.48±0.08	0.73±0.11	0.7±0.07	0.67±0.05	1±0	0.99±0.05
chrIV*	26,016,955	26,026,868	0.14±0.04	0.11±0.05	0.42±0.08	0.8±0.16	0.59±0.08	0.69±0.05	1±0.01	0.76±0.19
chrIV*	26,027,377	26,040,477	0.14±0.05	0.1±0.06	0.39±0.1	0.76±0.1	0.57±0.06	0.66±0.07	0.99±0.04	0.7±0.1
chrIV*	26,047,625	26,071,783	0.14±0.04	0.12±0.06	0.42±0.09	0.71±0.09	0.58±0.09	0.67±0.07	1±0	0.69±0.13
chrIV*	26,079,387	26,091,546	0.14±0.04	0.09±0.07	0.41±0.09	0.76±0.1	0.57±0.09	0.66±0.07	0.99±0.04	0.68±0.11
chrIV*	26,110,063	26,117,725	0.14±0.05	0.11±0.08	0.39±0.15	0.77±0.17	0.54±0.12	0.68±0.09	1±0.01	0.67±0.11

chrIV*	26,127,316	26,146,758	0.14±0.04	0.11±0.06	0.42±0.07	0.7±0.09	0.57±0.06	0.65±0.07	1±0.01	0.65±0.07
chrIV*	26,156,442	26,166,536	0.15±0.05	0.09±0.07	0.32±0.1	0.54±0.12	0.54±0.1	0.65±0.09	0.98±0.05	0.76±0.13
chrV	2,482,209	2,501,295	0.11±0.05	0.13±0.06	0.56±0.1	0.73±0.13	0.89±0.11	1±0	1±0.02	1±0
chrVII*	17,982,351	18,002,671	0.08±0.04	0.05±0.06	0.05±0.05	0.23±0.11	0.92±0.11	0.83±0.08	0.58±0.07	1±0
chrIX*	8,521,935	8,537,559	0.11±0.05	0.04±0.06	0.16±0.07	0.43±0.11	0.55±0.09	0.69±0.06	0.55±0.04	0.79±0.15
chrIX*	8,901,816	8,910,115	0.13±0.04	0.08±0.07	0.22±0.09	0.46±0.12	0.55±0.09	0.16±0.28	0.63±0.15	0.72±0.2
chrIX	9,208,158	9,227,809	0.14±0.06	0.05±0.06	0.41±0.09	0.66±0.09	0.75±0.1	0.67±0.06	0.97±0.05	1±0
chrIX	10,334,101	10,353,801	0.12±0.05	0.07±0.06	0.28±0.08	0.73±0.09	0.7±0.07	0.9±0.08	1±0.01	1±0.01
chrXI*	5,445,757	5,456,249	0.06±0.06	0.02±0.04	0.13±0.08	0.47±0.14	0.74±0.09	0.1±0.08	0.73±0.13	0.71±0.15
chrXI*	5,457,608	5,510,580	0.05±0.05	0.04±0.05	0.13±0.07	0.48±0.12	0.78±0.09	0.1±0.07	0.71±0.11	0.78±0.15
chrXI*	5,511,288	5,556,511	0.06±0.06	0.03±0.05	0.16±0.08	0.48±0.12	0.8±0.09	0.12±0.09	0.72±0.11	0.81±0.14
chrXI*	5,562,142	5,606,534	0.04±0.05	0.02±0.04	0.15±0.09	0.49±0.13	0.78±0.09	0.09±0.08	0.72±0.1	0.79±0.13
chrXI*	5,609,100	5,630,872	0.04±0.05	0.02±0.04	0.2±0.12	0.48±0.11	0.8±0.08	0.07±0.08	0.75±0.11	0.8±0.11
chrXI*	5,631,913	5,648,416	0.04±0.05	0.01±0.02	0.12±0.08	0.46±0.12	0.79±0.09	0.11±0.12	0.75±0.11	0.75±0.11
chrXI*	5,672,122	5,711,862	0.06±0.05	0.04±0.06	0.14±0.1	0.49±0.12	0.79±0.09	0.11±0.09	0.73±0.11	0.81±0.14
chrXI*	5,714,308	5,756,262	0.06±0.05	0.06±0.06	0.14±0.07	0.48±0.14	0.74±0.14	0.1±0.06	0.72±0.12	0.78±0.15
chrXI*	5,756,905	5,817,640	0.06±0.06	0.05±0.06	0.18±0.1	0.5±0.12	0.78±0.09	0.12±0.1	0.76±0.13	0.8±0.15
chrXI*	5,818,096	5,855,124	0.06±0.05	0.03±0.05	0.12±0.06	0.47±0.11	0.78±0.09	0.1±0.05	0.69±0.09	0.78±0.15

chrXII	14,338,229	14,358,336	0.15±0.04	0.1±0.06	0.12±0.06	0.41±0.11	0.84±0.11	0.83±0.08	1±0.02	1±0
chrXII	16,522,028	16,530,706	0.08±0.05	0.05±0.08	0.33±0.05	0.44±0.11	0.99±0.02	0.29±0.08	0.95±0.07	0.99±0.03
chrXII	16,537,728	16,538,810	0.07±0.07	0.06±0.07	0.32±0.09	0.37±0.08	0.98±0.03	0.28±0.03	0.93±0.08	0.99±0.02
chrXIX	2,449,903	2,508,206	0.13±0.04	0.07±0.06	0.36±0.1	0.93±0.13	0.76±0.12	0.69±0.08	1±0.01	1±0
chrXIX	2,543,385	2,553,623	0.12±0.07	0.06±0.08	0.39±0.16	0.91±0.15	0.69±0.06	0.64±0.11	0.99±0.05	0.99±0.04
chrXIX	2,573,853	2,581,858	0.1±0.06	0.06±0.06	0.28±0.08	0.88±0.15	0.69±0.07	0.58±0.08	1±0	1±0
chrXIX	14,787,904	14,799,088	0.09±0.06	0.05±0.06	0.24±0.07	0.49±0.18	0.61±0.12	0.37±0.13	0.99±0.04	0.98±0.05
chrXXI*	5,759,879	5,776,968	0.04±0.06	0.02±0.05	0.28±0.07	0.29±0.12	0.46±0.12	0.24±0.22	0.7±0.11	0.6±0.1
chrXXI*	5,790,637	5,816,820	0.01±0.02	0.05±0.06	0.36±0.1	0.59±0.13	0.47±0.08	0.01±0.05	0.66±0.08	0.58±0.07
chrXXI*	5,817,730	5,829,114	0±0.01	0.02±0.04	0.35±0.09	0.55±0.16	0.47±0.1	0±0	0.68±0.09	0.59±0.08
chrXXI*	5,835,764	5,914,891	0±0.02	0.03±0.05	0.36±0.09	0.57±0.13	0.47±0.1	0±0.03	0.7±0.1	0.58±0.08
chrXXI*	5,919,392	5,929,442	0.01±0.02	0.02±0.03	0.34±0.06	0.55±0.11	0.44±0.09	0±0	0.71±0.07	0.57±0.05
chrXXI*	5,930,066	5,939,357	0.01±0.04	0.03±0.05	0.37±0.09	0.59±0.15	0.42±0.09	0.02±0.04	0.65±0.13	0.66±0.19
chrXXI*	5,940,493	5,981,085	0±0.02	0.03±0.05	0.36±0.1	0.6±0.12	0.46±0.11	0.01±0.03	0.71±0.14	0.59±0.11
chrXXI*	5,982,524	6,116,717	0.01±0.02	0.03±0.05	0.36±0.1	0.59±0.12	0.47±0.09	0.01±0.05	0.69±0.1	0.6±0.08
chrXXI*	6,117,537	6,148,626	0±0.01	0.02±0.04	0.35±0.09	0.57±0.11	0.47±0.09	0.01±0.05	0.69±0.14	0.59±0.08
chrXXI*	6,152,036	6,160,856	0±0.02	0.02±0.05	0.37±0.05	0.67±0.14	0.48±0.13	0.03±0.12	0.75±0.1	0.6±0.11
chrXXI*	6,162,338	6,387,109	0±0.02	0.03±0.05	0.35±0.09	0.59±0.12	0.47±0.1	0.01±0.05	0.69±0.1	0.58±0.08

chrXXI*	6,387,893	6,401,909	0.01±0.02	0.02±0.04	0.35±0.07	0.58±0.1	0.48±0.08	0±0.01	0.66±0.09	0.55±0.08
chrXXI*	6,415,345	6,625,069	0.01±0.02	0.03±0.05	0.36±0.1	0.57±0.15	0.47±0.12	0.01±0.04	0.69±0.11	0.59±0.09
chrXXI*	6,627,144	6,644,932	0.01±0.01	0.04±0.06	0.38±0.11	0.6±0.14	0.47±0.1	0.03±0.11	0.71±0.14	0.59±0.07
chrXXI*	6,645,868	6,675,900	0±0.02	0.02±0.05	0.36±0.08	0.6±0.13	0.46±0.11	0.01±0.04	0.65±0.1	0.59±0.09
chrXXI*	6,677,055	6,806,865	0±0.02	0.03±0.05	0.35±0.1	0.6±0.13	0.46±0.1	0.01±0.05	0.68±0.11	0.58±0.08
chrXXI*	6,811,271	6,822,421	0.01±0.01	0.02±0.04	0.34±0.12	0.54±0.1	0.46±0.08	0.02±0.09	0.68±0.11	0.58±0.07
chrXXI*	6,822,926	6,838,750	0±0.02	0.03±0.05	0.33±0.07	0.57±0.1	0.44±0.1	0±0	0.65±0.09	0.58±0.07
chrXXI*	6,840,532	6,849,088	0±0.01	0.02±0.04	0.32±0.1	0.55±0.1	0.46±0.07	0±0.01	0.65±0.1	0.55±0.07
chrXXI*	6,853,153	6,885,035	0±0.01	0.02±0.04	0.35±0.1	0.58±0.13	0.46±0.11	0±0.01	0.7±0.11	0.58±0.08
chrXXI*	6,885,335	6,895,947	0.02±0.05	0.04±0.05	0.35±0.07	0.58±0.14	0.47±0.12	0.02±0.07	0.71±0.13	0.61±0.06
chrXXI*	6,897,734	6,949,514	0±0.02	0.02±0.04	0.39±0.1	0.62±0.15	0.46±0.11	0±0.01	0.69±0.11	0.61±0.1
chrXXI*	6,952,798	6,988,645	0.01±0.03	0.03±0.05	0.36±0.1	0.61±0.13	0.5±0.1	0.03±0.13	0.69±0.12	0.58±0.07
chrXXI*	6,990,542	6,999,498	0±0.01	0.02±0.04	0.34±0.08	0.61±0.15	0.45±0.1	0±0	0.68±0.13	0.6±0.1
chrXXI*	7,001,769	7,013,989	0.01±0.03	0.03±0.04	0.35±0.11	0.61±0.07	0.5±0.09	0±0	0.68±0.07	0.57±0.06
chrXXI*	7,014,476	7,036,127	0.01±0.03	0.03±0.05	0.36±0.09	0.57±0.11	0.45±0.09	0±0	0.67±0.1	0.57±0.06
chrXXI*	7,037,602	7,087,560	0±0.01	0.03±0.05	0.35±0.08	0.61±0.12	0.47±0.08	0±0.01	0.67±0.09	0.59±0.09
chrXXI*	7,092,045	7,191,351	0±0.02	0.04±0.05	0.37±0.1	0.61±0.12	0.5±0.1	0±0.01	0.71±0.13	0.59±0.09
chrXXI*	7,194,393	7,212,749	0±0.02	0.04±0.05	0.38±0.1	0.6±0.1	0.52±0.1	0.01±0.06	0.7±0.1	0.59±0.09

chrXXI*	7,214,239	7,486,635	0±0.02	0.03±0.05	0.35±0.09	0.59±0.12	0.47±0.1	0.01±0.04	0.69±0.11	0.59±0.08

Table S7. Mean frequencies of freshwater alleles in each population for DIs found in our study without merging procedure, mean ± st. dev.
